# Molecular Analysis and Phenotypic Study in 14 Chinese Families with Bietti Crystalline Dystrophy

**DOI:** 10.1371/journal.pone.0094960

**Published:** 2014-04-16

**Authors:** Houfa Yin, Chongfei Jin, Xiaoyun Fang, Qi Miao, Yingying Zhao, Zhiqing Chen, Zhaoan Su, Panpan Ye, Yao Wang, Jinfu Yin

**Affiliations:** 1 Eye Center, Second Affiliated Hospital of Zhejiang University School of Medicine, Hangzhou, Zhejiang Province, China; 2 Zhejiang Provincial Key Laboratory of Ophthalmology, Hangzhou, Zhejiang Province, China; 3 Ophthalmic Genetics and Visual Function Branch, National Eye Institute, NIH, Bethesda, Maryland, United States of America; Innsbruck Medical University, Austria

## Abstract

**Purpose:**

To investigate the clinical features and cytochrome P450 family 4 subfamily V polypeptide 2 (*CYP4V2*) gene mutations in 14 Chinese families with Bietti crystalline dystrophy (BCD).

**Methods:**

Seventeen patients from 14 unrelated Chinese families with BCD were recruited for complete clinical ophthalmic examination and genetic study. The 11 exons of *CYP4V2* were amplified from genomic DNA of all patients and their family members by polymerase chain reaction (PCR) and then sequenced. Exons of *TIMP3* were also sequenced in BCD patient associated with choroidal neovascularization (CNV). One hundred and seventy unrelated healthy Chinese subjects were screened for mutations in *CYP4V2*.

**Results:**

All 17 patients with BCD had mutations in *CYP4V2*; one of these mutations was novel (c.219T>A, p.F73L) and four other mutations had been reported. The p.F73L mutation was a commonly detected mutation in our study (seven out of 34 alleles), either in the homozygous state or in the heterozygous state. Among the patients, considerable phenotypic variability was detected, both within and between families. Screening of *TIMP3* did not find any mutation in the BCD patient associated with CNV.

**Conclusion:**

The novel *CYP4V2* c.219T>A (p.F73L) mutation may be another recurrent mutation in Chinese patients with BCD. Our study expands the mutation spectrum of *CYP4V2* and characterizes novel genotype–phenotype associations in Chinese patients with BCD.

## Introduction

Bietti crystalline dystrophy (BCD, MIM 210370), first described by Bietti in 1937 [1], is a rare progressive chorioretinal degenerative disease characterized by numerous yellow-white dot crystalline deposits scattered over the fundus, and is associated with atrophy of the retinal pigment epithelium (RPE) and the choriocapillaris, pigment clumping, and choroidal sclerosis, with or without the presence of limbal corneal crystals [2], [3]. Although corneal crystalline dystrophy in this disorder is characterized by the presence of glistening limbic deposits visible in 360 degrees and mainly presents in Caucasians [4], [5], [6], central and paracentral corneal distribution of the crystalline deposits without limbic involvement has also been reported recently [7], suggesting significant phenotypic variations of this disorder. BCD is a progressive disease characterized by nyctalopia, decreased visual acuity, and paracentral scotoma between the second and fourth decades of life, progressing to marked visual impairment, visual field constriction, and legal blindness by the fifth or sixth decades of life [7], [8].

BCD is a rare autosomal recessive disease, which accounts for 3% of all non-syndromic retinitis pigmentosa cases and 10% of autosomal recessive retinitis pigmentosa cases [6]. The causative gene for BCD, *CYP4V2* (MIM 608614), has been identified in chromosome 4q35.1 by Li and colleagues [2], [9]. To date, more than 50 mutations of *CYP4V2* have been identified in patients with BCD [8], [10], [11], [12], [13], [14], [15], [16]. CYP4V2 is a member of the cytochrome P450 heme thiolate protein superfamily and may be the dominant functional CYP4 enzyme in disease-targeted ocular tissues of BCD, such as RPE and corneal epithelium [17]. It plays a key role in modulating the metabolism of fatty acids/lipids in the eye, especially ω-3 polyunsaturated fatty acids (PUFAs) [17]. Recent studies suggest that defective ω-oxidation of ocular fatty acids/lipids secondary to mutations in *CYP4V2* may contribute to the abnormal lipid metabolism in BCD patients [17], [18], [19], [20].

Choroidal neovascularization (CNV) usually occurs in individuals with defects in RPE and Bruch's membrane, such as age-related macular degeneration (AMD) and pathologic myopia. However, CNV infrequently occurs in patients affected by hereditary retinal dystrophies [21]. To date, there are only four reports of BCD associated with the phenotype of CNV [14], [22], [23], [24],whereas none of them identified causative mutations in *CYP4V2* or other genes associated with CNV, such as tissue inhibitor of metalloproteinase 3 (*TIMP3*) [14], [25]. In addition, the phenotype of BCD associated with a macular hole was only briefly described in two studies without genetic analysis [26], [27].

In this study, we sought to investigate the clinical features and *CYP4V2* mutations in 17 Chinese patients from 14 unrelated families with BCD. We identified five mutations in *CYP4V2*, and characterized the clinical and genetic features of two atypical forms of BCD, including BCD associated with CNV and BCD associated with a macular hole.

## Materials and Methods

### Ethics Statement

The study protocol was approved by the Institutional Review Board of Second Affiliated Hospital of Zhejiang University School of Medicine and adhered to the tenets of the Declaration of Helsinki. Written informed consent was obtained from all the participants.

### Patients

All patients and their family members were recruited from the Eye Center of Second Affiliated Hospital, Medical College of Zhejiang University, Hangzhou, China. All participants underwent a complete eye examination including best correct visual acuity (BCVA) according to projected Snellen charts, slit-lamp biomicroscopy, applanation tonometry, fundus examination, and fundus photography. Among them, 14 patients underwent fundus fluorescein angiography (Heidelberg Engineering HRA Spectralis, Heidelberg, Germany), 11 patients underwent spectral domain optical coherence tomography (Zeiss Cirrus OCT, Carl Zeiss, Dublin, CA), and 7 patients underwent static visual field tests using Octopus perimetry (Octopus; Interzeag, Schlieren, Switzerland). BCD was diagnosed according to the symptoms, typical fundus findings, and clinical examinations based on the stage classification of Yuzawa et al. [28]. As recommended by Collins and Schwartz [29], 170 unrelated healthy subjects were also recruited as controls to distinguish the predicted mutation, with 95% confidence, from a polymorphism with 1% frequency. Both the participants and the controls were from a Chinese Han population in Southeastern China.

### Mutation Screening and Sequence Analyses

Blood samples of all participants were drawn by venipuncture and collected in Vacutainer tubes (Becton-Dickinson, Franklin Lakes, NJ) containing ethylene diamine tetraacetic acid (EDTA). Genomic DNA was extracted using the Simgen Blood DNA mini kit (Hangzhou Simgen Biotechnology Co. Ltd, Hangzhou, China), according to the manufacturer's recommendations. All 11 coding exons with flanking splice junctions of the *CYP4V2* gene (NM_207352.3) were amplified by polymerase chain reaction (PCR) using primers published previously [2]. The PCR products were purified and sequenced using the BigDye Terminator Cycle sequencing kit V 3.1 (ABI-Applied Biosystems, Sangon Co, China) on an ABI PRISM 3730 Sequence Analyzer (ABI), according to the manufacturer's instructions. Sequencing results were analyzed using Chromas 1.62 and compared with sequences from the NCBI GenBank (*CYP4V2*, NM_207352.3). In BCD patient associated with CNV, exons with flanking splice junctions of the *TIMP3* gene (NM_000362.4) were also amplified via PCR using primers published previously and sequenced [30]. Each variation observed was confirmed by bidirectional sequencing and then evaluated in 170 controls. The effect of mutations on protein function was estimated by PROVEAN (http://provean.jcvi.org/index.php) and Polyphen-2 (http://genetics.bwh.harvard.edu/pph2/), and conservation of the affected amino acid across species was analyzed using Tcoffee (http://igs-server.cnrs-mrs.fr/Tcoffee/tcoffee_cgi/index.cgi).

## Results

A total of 17 BCD patients (8 males, 9 females, mean age, 36.4±9.3 years, range, 24–60 years) from 14 unrelated families were enrolled in this study. Among the patients, there were three groups of siblings (patients P6.1, P6.2, P11.1, P11.2, P12.1 and P12.2). In addition, three patients (P1, P12.1 and P12.2) belonged to consanguineous families whose parents were first cousins. Clinical and genetic findings of the patients are described in Tables 1 and 2.

**Table 1:** Summary of clinical and genetic features in patients with *CYP4V2* mutations

Family No.	Patient No.	Sex	Age (years)	BCVA		Mutation	
				RE	LE	Allele1	Allele2
F1	P1	F	39	0.04	0.8	c.283G>A	c.802-8_810del17insGC
F2	P2	F	47	0.8	0.6	c.802-8_810del17insGC	c.802-8_810del17insGC
F3	P3	M	37	0.08	0.2	c.802-8_810del17insGC	c.1091-2A>G
F4	P4	F	42	0.3	0.4	c.802-8_810del17insGC	c.802-8_810del17insGC
F5	P5	F	34	1.5	1.5	c.802-8_810del17insGC	c.802-8_810del17insGC
F6	P6.1	M	27	0.1	0.3	c.802-8_810del17insGC	c.1091-2A>G
F6	P6.2	F	28	0.8	1.0	c.802-8_810del17insGC	c.1091-2A>G
F7	P7	F	24	0.6	0.6	c.219T>A	c.219T>A
F8	P8	M	33	0.8	0.05	c.802-8_810del17insGC	c.1091-2A>G
F9	P9	M	32	1.0	FC	c.1091-2A>G	c.1062dupA
F10	P10	M	32	0.3	0.05	c.219T>A	c.802-8_810del17insGC
F11	P11.1	F	34	0.3	0.3	c.1091-2A>G	c.1091-2A>G
F11	P11.2	M	40	0.5	0.3	c.1091-2A>G	c.1091-2A>G
F12	P12.1	F	60	0.8	FC	c.219T>A	c.219T>A
F12	P12.2	M	50	0.8	0.7	c.219T>A	c.219T>A
F13	P13	F	28	0.6	0.6	c.802-8_810del17insGC	c.1091-2A>G
F14	P14	M	31	0.4	0.1	c.802-8_810del17insGC	c.802-8_810del17insGC

BCVA, best corrected visual acuity; RE, right eye; LE, left eye; M, male; F, female; FC, finger count.

**Table 2:** Summary of clinical features in patients with *CYP4V2* mutations

Family No.	Patient No.	Fundus changes
F1	P1	BE: RA with CD at the posterior pole and midperiphery, CA at the posterior pole
F2	P2	BE: RA and CA with CD at the posterior pole and midperiphery
F3	P3	BE: Extensive RA and CA at the posterior pole and midperiphery, CD at the midperiphery, RVA
F4	P4	BE: RA and CA with CD and PC at the posterior pole and midperiphery
F5	P5	BE: RA and CA with CD and PC at the posterior pole and midperiphery
F6	P6.1	BE: RA with CD at the posterior pole and midperiphery, CA at the posterior pole; RE: macular hole
F6	P6.2	BE: RA with CD at the posterior pole and midperiphery, CA at the posterior pole
F7	P7	BE: RA with CD at the posterior pole and midperiphery, CA at the posterior pole
F8	P8	BE: RA and CA with CD and PC at the posterior pole and midperiphery
F9	P9	BE: RA with CD at the posterior pole and midperiphery; LE: CNV at the macular and PC
F10	P10	BE: Extensive RA and CA with CD and PC at the posterior pole and midperiphery
F11	P11.1	BE: RA and CA with CD at the posterior pole and midperiphery
F11	P11.2	BE: RA and CA with CD at the posterior pole and midperiphery
F12	P12.1	BE: RA and CA with CD and PC at the posterior pole and midperiphery
F12	P12.2	BE: Extensive RA and CA with CD at the posterior pole and midperiphery
F13	P13	BE: RA with CD at the posterior pole and midperiphery, CA at the posterior pole
F14	P14	BE: RA and CA with CD at the posterior pole and midperiphery

BE, both eyes; RE, right eye; LE, left eye; CD, crystalline deposits; RA, retinal pigment epithelium atrophy; CA, choriocapillaris atrophy; RVA, retinal vascular attenuation; PC, pigment clumping.

### Mutation Identification and Analysis

**Figure 1:**
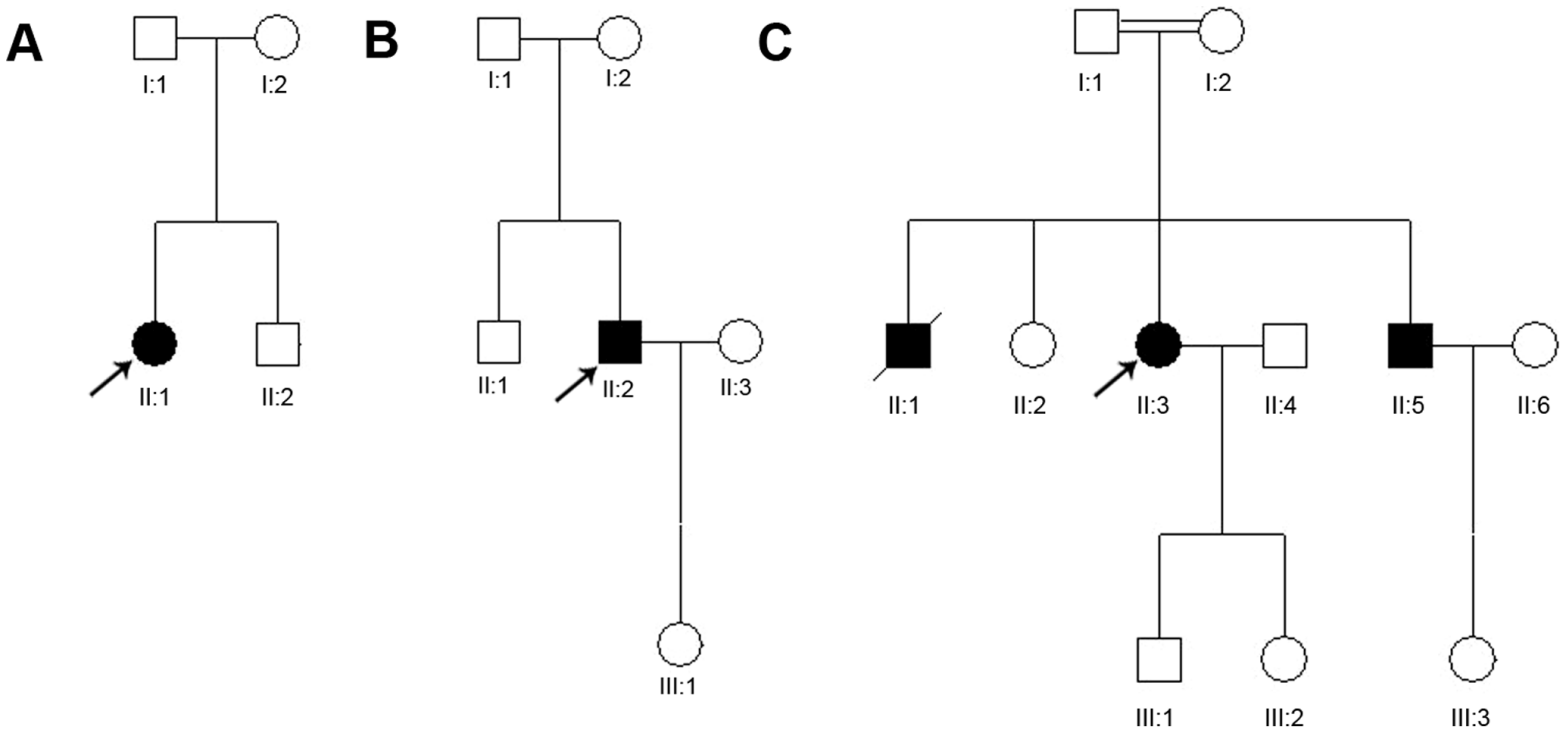
Pedigree of the three families in this study with novel *CYP4V2* mutation c.219T>A (p.F73L). (A) Pedigree of family 7 (F7 in Table 1). (B) Pedigree of family 10 (F10 in Table 1). (C) Pedigree of family 12 (F12 in Table 1). Squares, males; circles, females; filled squares or circles, affected family members; slash, deceased family member; arrow, proband; asterisk, a study participant from whom a blood sample was obtained.

**Figure 2:**
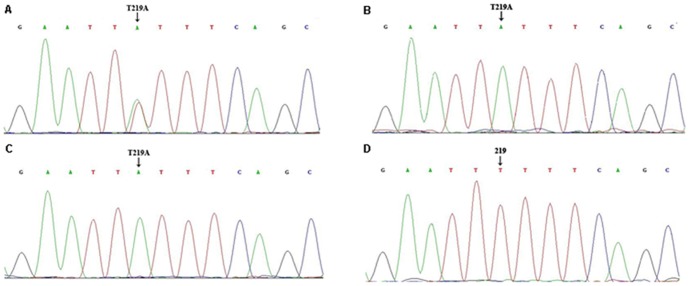
Identification of the *CYP4V2* c.219T>A mutation. Partial DNA sequence of *CYP4V2* exon 2 in DNA from three probands and a control subject. (A) Arrow shows a heterozygous T to A transition at nucleotide position 219 in patient P10. (B) and (C) Arrow shows a homozygous T to A transition at nucleotide position 219 in patient P7 and patient P12.1, respectively. (D) Arrow shows normal base at nucleotide position 219 in the control subject.

**Figure 3:**
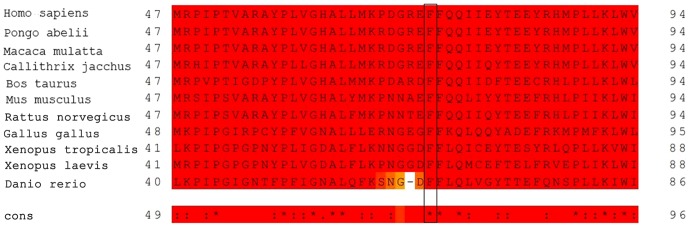
Multiple sequence alignment for CYP4V2 (partially). The phenylalanine residue at position 73 (rectangle) is highly conserved across primates and other vertebrates.

After sequencing the coding exons with flanking splice junctions of *CYP4V2*, sequence variants were identified in all 17 patients from 14 unrelated families, including c.219T>A (p.F73L), c.283G>A (p.G95R), c.802-8_810del17insGC (splicing site), c.1062dupA (p.V355Sfs*4), and c.1091-2A>G (splicing site) (Table 1). Of the five sequence variants, c.283G>A, c.802-8_810del17insGC, c.1062dupA, and c.1091-2A>G have been reported previously; c.219T>A was detected for the first time. The most common mutation observed was c.802-8_810del17insGC found in 15 (44%) of the 34 mutant alleles; however, c.1091-2A>G and c.219T>A were also frequent, which were found in 10 (29.4%) and 7 (20.6%) of the 34 mutant alleles, respectively. The novel sequence variant c.219T>A (p.F73L) was identified in the homozygous state in three patients (P7, P12.1 and P12.2) and was present in the heterozygous state associated with c.802-8_810del17insG in patient P10 (Table 1, Figures 1 and 2). This sequence variant results in a substitution of phenylalanine with leucine. PROVEAN predicted that the variant was deleterious with a score of -2.773, whereas Polyphen-2 predicted this substitution was benign. The phenylalanine at position 73 is well conserved across primates and other vertebrates as demonstrated in Figure 3. Furthermore, this change was absent in 340 control alleles, and the patients were not identified as harboring any other mutations in *CYP4V2*. Therefore, we propose that p.F73L is classified as pathogenic. In BCD patient associated with unilateral CNV (patient P9), we identified previously reported compound heterozygous mutations, a frequent splice site mutation c.1091-2A>G, and a newly detected single base-pair duplication mutation c.1062dupA [14]. However, no pathogenic variant was observed in *TIMP3* in this patient. In addition, compound heterozygous mutations c.[802-8_810del17insGC];[1091-2A>G] were identified in patient P6.1, who was associated with a unilateral lamellar hole.

### Clinical Characteristics

Patients experienced night blindness or had a progressive decrease in visual acuity between the second and third decades of life. BCVA (decimal Snellen) ranged widely from finger count to completely normal. Intriguingly, BCVA was 1.5 in both eyes in patient P5, whereas she had mild night blindness that appeared in the third decade of life. Marked asymmetry between eyes in visual acuity was common in this study. In total, four patients (4/17, 23.5%) had marked asymmetry in visual acuity between eyes (≥4 lines). BCVA at the first clinical assessment in the right eye was 0.04, 0.8, 1.0, and 0.8, and in the left eye was 0.8, 0.05, counting fingers, and counting fingers in patients P1, P8, P9, and P12.1, respectively. Patient P9, with finger count vision in the right eye, was associated with CNV in this eye; whereas it was proposed that the worse visual acuity in the fellow eye in three other patients was mainly attributed to the more severe central macular involvement compared with the eye with better visual acuity.

**Figure 4:**
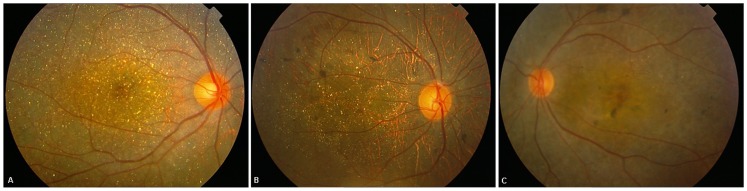
Fundus manifestations of three probands with the novel *CYP4V2* mutation (p.F73L) identified in this study. (A) Right eye of patient P7. (B) Right eye of patient P10. (C) Left eye of patient P12.1.

**Figure 5:**
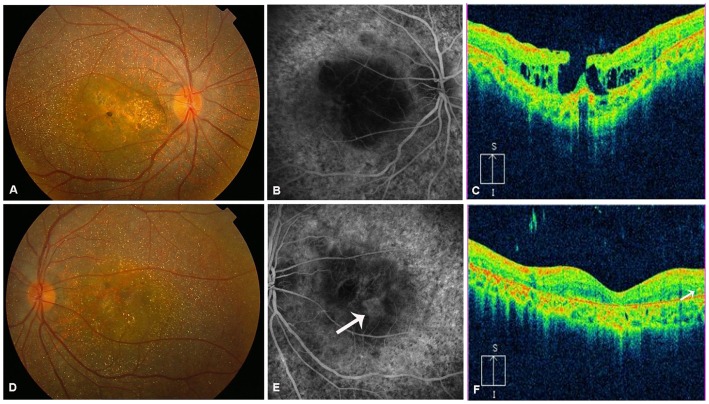
Ocular phenotype of patient P6.1. (A) Fundus photograph of right eye shows crystalline deposits with retinal pigment epithelium (RPE) and choriocapillaris atrophy and macular hole. (B) Late phase of fundus fluorescein angiography (FFA) of right eye shows choriocapillaris-RPE complex atrophy, while macular leakage is not shown. (**C**) Spectral domain-optical coherence tomography (SD-OCT) of right eye shows a lamellar hole attributable to macular cysts. (D) Fundus photograph of left eye shows crystalline deposits with RPE and choriocapillaris atrophy. (E) Late phase of FFA of left eye shows choriocapillaris-RPE complex atrophy, while partial reservation of choriocapillaris in macular is noted (white arrow). (F) SD-OCT of left eye shows thinning of the neuroretina, disturbed organization of the RPE-photoreceptor outer/inner segment layer, increased backscatter, and intraretinal hyperrefractive spot related to crystalline deposit (white arrow).

**Figure 6:**
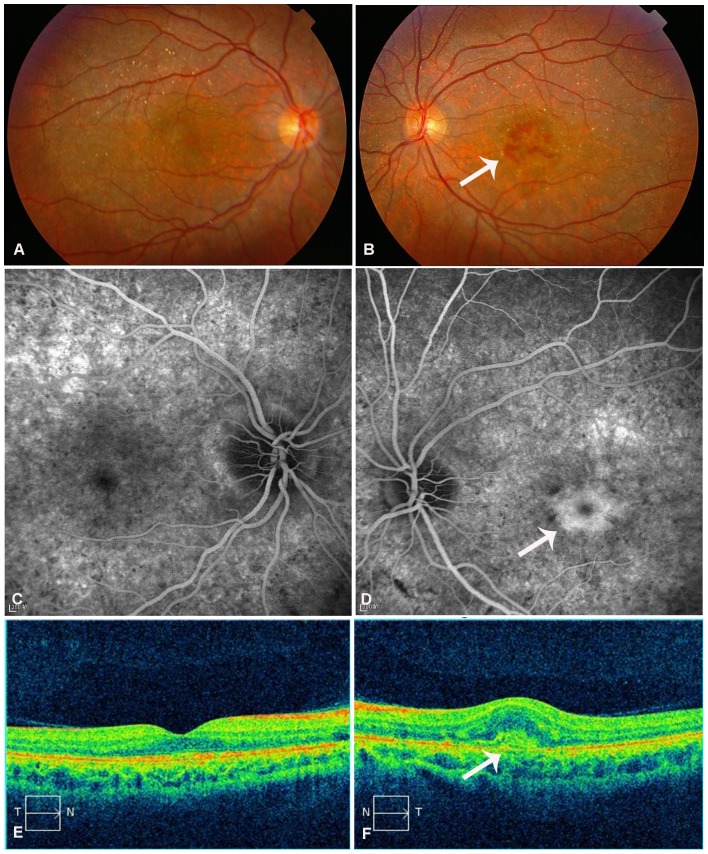
Ocular phenotype of patient P9 at initial examination. (A) Fundus photograph of right eye shows crystalline deposits with retinal pigment epithelium (RPE) atrophy. (B) Fundus photograph of left eye shows crystalline deposits with RPE atrophy, active choroidal neovascularization (CNV), and subretinal hemorrhage (white arrow). (C) Late phase of fundus fluorescein angiography (FFA) of right eye shows scattered hypofluorescent areas due to choriocapillaris filling defects, surrounded by areas of hyperfluorescent RPE window defects. (D) FFA of left eye shows central hyperfluorescence (leaked fluorescein) in the late phase, suggestive of active CNV. (E) Spectral domain-optical coherence tomography (SD-OCT) of right eye shows disturbed organization of the RPE-photoreceptor outer/inner segment layer. (F) SD-OCT of left eye shows a subfoveal hyperreflective pre-epithelial lesion and macular edema, suggestive of active CNV.

**Figure 7:**
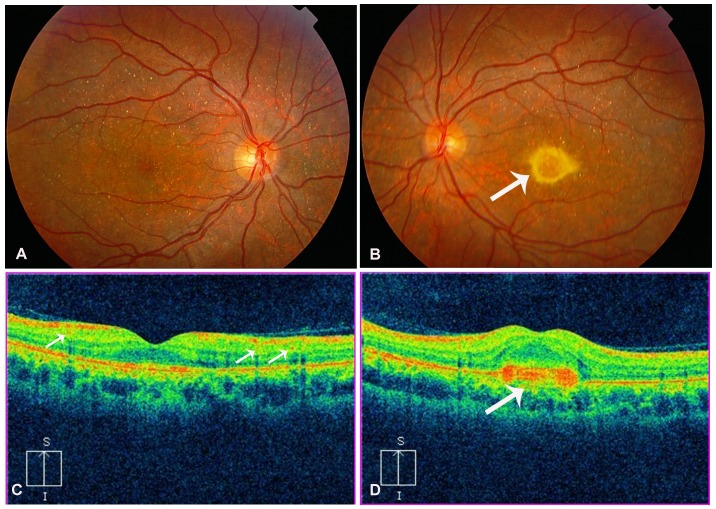
Ocular phenotype of patient P9 seven months later. (A) Fundus photograph of right eye shows crystalline deposits with retinal pigment epithelium (RPE) atrophy. (B) Fundus photograph of left eye shows scarred choroidal neovascularization (CNV, white arrow). (C) Spectral domain-optical coherence tomography (SD-OCT) of right eye shows intraretinal hyperreflective spots related to crystalline deposits (white arrows). (D) SD-OCT of left eye shows hyperreflective pre-epithelial lesion without intra- or subretinal fluid corresponding to scarred CNV (white arrow).

Slit-lamp examination in all patients revealed normal anterior segments, and corneal limbal crystals were not detected in any of the BCD patients. All patients with *CYP4V2* mutations in this study showed characteristic fundus manifestations of BCD, including numerous yellow-white dots crystalline deposits distributed on the posterior pole and mid-periphery, pigment deposits, retinal vascular attenuation, diffuse RPE atrophy and choriocapillaris atrophy, except in two patients (P6.1 and P9) in whom atypical forms of BCD fundus changes were noted. However, fundus manifestations of three probands with the novel p.F73L mutation were typical (Tables 1 and 2, Figure 4). In total, two atypical forms of BCD, including BCD associated with CNV and BCD associated with a macular hole, were found in our study. Harboring previously reported compound heterozygous mutations c.[802-8_810del17insGC];[1091-2A>G], patient P6.1 had a lamellar hole due to macular cysts in the right eye, while no similar macular hole was noted in his left eye (Figure 5, A and D). He was diagnosed at age 27 as he noticed a marked decrease in visual acuity in the right eye during the past year. His BCVA was 0.1 (right eye) and 0.3 (left eye). Macular hole was not found in an affected sibling (patient P6.2) who carried the same mutations. Patient P9 harboring compound heterozygous mutations c.[1091-2A>G];[1062dupA] diverted from characteristic fundus manifestations of BCD, as he presented with subretinal hemorrhage with active CNV in the subfoveal region of the left eye (Figure 6B); however, CNV was not noted in his right eye (Figure 6A). He experienced a sudden decrease in visual acuity in the left eye in the last month, with visual acuity 1.0 in the right eye and finger count in the left eye. Seven months later, visual acuity improved to 0.25 in the left eye and 1.0 was maintained in the right eye without any treatment. Fundus examination showed scarring from CNV in the left eye (Figure 7B) that was confirmed on spectral domain optical coherence tomography (SD-OCT) (Figure 7D).

**Figure 8:**
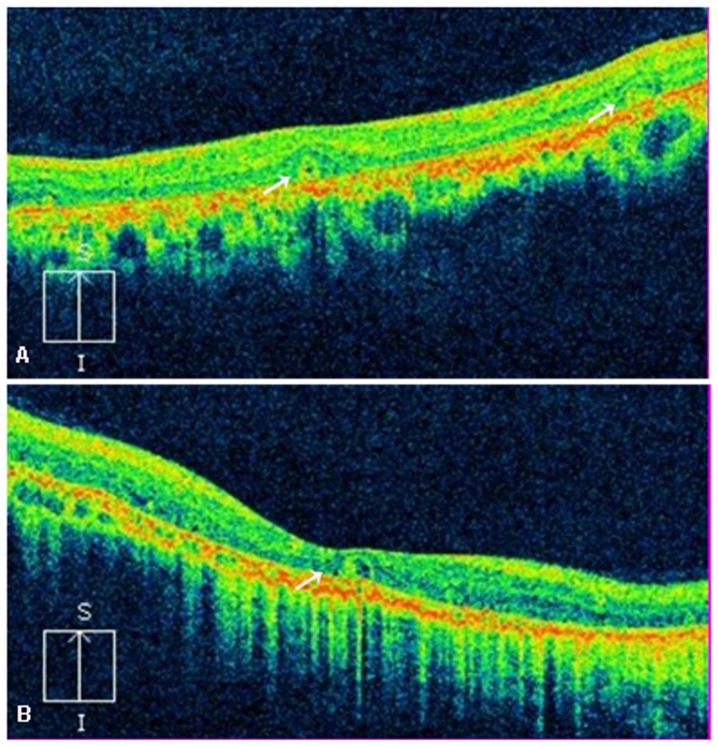
Spectral domain-optical coherence tomography (SD-OCT) image of BCD with outer retinal tubulation (ORT, white arrows). (A) SD-OCT of right eye of patient P1. (B) SD-OCT of right eye of patient P3.

**Figure 9:**
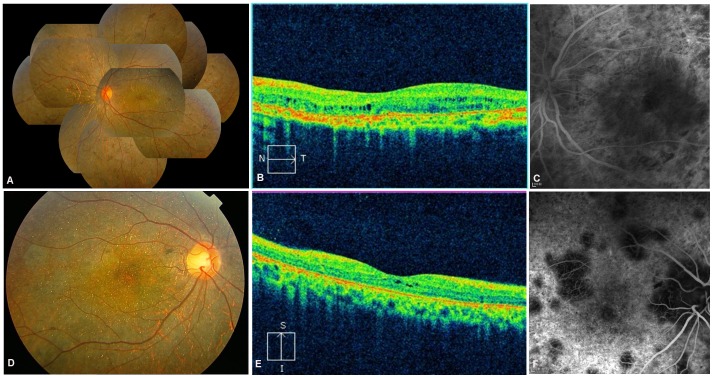
Ocular phenotype of patients with microcystic changes in the neuroretina. (A) Fundus photograph of left eye of patient P5. (B) Spectral domain-optical coherence tomography (SD-OCT) of left eye of patient P5 shows multiple microcystic changes in multiple retinal layers. (C) Late phase of fundus fluorescein angiography (FFA) of left eye of patient P5 shows no fluorescein leakage. (D) Fundus photograph of right eye of patient P2. (E) SD-OCT of right eye of patient P2 shows subfoveal microcystic changes. (F) Late phase of FFA of right eye of patient P2 shows no fluorescein leakage as well.

Eleven patients underwent SD-OCT examinations of the macula, which showed the presence of diffuse intraretinal hyperrefractive spots related to crystalline deposits within the neuroretinal layers and disturbed organization of the RPE-photoreceptor outer/inner segment layer in all patients. In addition, SD-OCT revealed thinning of the retina at the central macular region outside the 5% limit of the normal population in 13 (65%) of the 22 eyes, while outer retinal tubulation (ORT) was only noted in the retina in two patients (P1 and P3, Figure 8). Furthermore, SD-OCT analysis disclosed details in the structural changes of the retina corresponding to patient P6.1 and patient P9. SD-OCT in patient P6.1 revealed a lamellar hole attributable to macular cysts in the left eye; however, there were no cystoid lesions in the macula in his right eye (Figure 5, C and F). SD-OCT revealed the presence of a subfoveal hyperreflective pre-epithelial lesion and macular edema suggestive of active CNV in the left eye in patient P9 (Figure 6F). Seven months later, SD-OCT showed a hyperreflective pre-epithelial lesion without intra- or subretinal fluid corresponding to scarred CNV in patient P9 (Figure 7D). SD-OCT also disclosed multiple microcystic changes in multiple retinal layers in two patients, which were identified bilaterally in patient P5 with predominance in the left eye and unilaterally in patient P2 (Figure 9, B and E).

Fundus fluorescein angiography (FFA) performed on 14 patients showed varying degrees of choriocapillaris-RPE complex atrophy and choriocapillaris non-perfusion ranging from scattered hypofluorescent areas surrounded by areas of hyperfluorescent RPE window defects in the posterior pole to the totally atrophy of the whole choriocapillaris-RPE complex. Also, FFA performed on patient P9 showed central hyperfluorescence in the early phase and leakage in the late phase in the left eye corresponding to active CNV (Figure 6D). Intriguingly, discordance was noted between findings on the FFA and SD-OCT either in patient P6.1 with macular cysts resulting in a macular hole or in patients P2 and P3 with microcystic changes. In these patients, SD-OCT disclosed the presence of cystoid lesions in the macula, but there was no dye accumulation on the FFA (Figure 5, B and C; Figure 9, B and C; Figure 9, E and F).

Visual field testing performed on seven patients showed varying degrees of central visual field loss in both eyes including central, paracentral or arcuate scotoma, partial or complete ring scotoma and tubular fields (further data is available upon request).

## Discussion

In this study, we assessed the genotypes and phenotypes of 17 patients from 14 unrelated Chinese families with BCD. All the BCD patients harbored *CYP4V2* mutations, and gene sequencing identified five mutations, including a novel mutation c.219T>A (p. F73L) and four known mutations, c.283G>A (p.G95R), c.802-8_810del17insGC (splicing site), c.1062dupA (p.V355Sfs*4), and c.1091-2A>G (splicing site). Among these patients, we also identified two patients with atypical phenotypes including BCD associated with CNV in patient P9 and BCD associated with a macular hole in patient P6.1. To our knowledge, this is the first report of BCD associated with CNV or a macular hole caused by mutations in *CYP4V2*.

The *CYP4V2* gene, located in chromosome 4q35.1, consists of 11 exons that encode a predicted protein of 525 amino acids [2]. CYP4V2 is a member of the cytochrome P450 (P450) heme thiolate protein superfamily and is termed as an “orphan” P450 because its substrate specificity and physiological roles are just beginning to be clarified [17], [19]. According to the homology modeling, the predicted CYP4V2 protein structure consists of a transmembrane segment at the N-terminus, followed by a globular structural domain typical of P450s [2]. Just as other well characterized CYP4 enzymes, CYP4V2 possesses typical CYP4 ω-hydroxylase activity and plays a significant role in fatty acid homeostasis especially in the eye [19]. Nakano et al.[17] recently demonstrated that CYP4V2 was the most highly expressed P450 mRNA in ARPE-19 cells, naturally transformed human RPE cells, and the CYP4V2 protein was heavily expressed in human retinal epithelium, and to a lesser extent, in corneal epithelium. Therefore, CYP4V2 is the major PUFAs hydroxylase in BCD target tissues in the eye, and the p.H331P mutation of CYP4V2 was reported to result in significant decreases in the total levels of several PUFAs in the HepG2 cells [17].

To date, 58 distinct mutations have been identified (see Table S1). Of these mutations, c.802-8_810del17insGC was the most common mutation, accounting for 62.7% of all mutant alleles [8]. Consistent with previous studies, c.802-8_810del17insGC was found to be the most common mutation in our study (present in 15 out of the 34 mutant alleles). However, the novel mutation c.219T>A (p.F73L) was also frequently detected in our study, 7 (23.3%) out of the 34 mutant alleles, either in the homozygous state or in the heterozygous state. These results suggested that the p.F73L mutation should be one of the first targets in mutation based diagnosis and gene based therapy for BCD.

Although a previous report has demonstrated that BCD was a clinically and genetically homogeneous disease [8], considerable inter- and intrafamilial phenotypic variations were noted in our study even with the same genotype as described in our studies (Tables 1 and 2). For example, harboring the same homozygous mutation c.802-8_810del17insGC, patient P5 and patient P14 showed a high variation with BCVA, 1.5 in patient P5 versus less than 0.5 in patient P14. In addition, patient P6.2 retained good visual acuity, whereas her sibling, patient P6.1 harboring the same mutations presented a lamellar hole in the unilateral eye with worse VA. The lack of a clear genotype–phenotype correlation might be attributed to other modifying or environmental factors, as suggested previously [12], [31].

Compound heterozygous mutations c.[1091-2A>G];[1062dupA] were detected in a BCD patient associated with unilateral subfoveal CNV, which was confirmed by SD-OCT and FFA. The splicing acceptor site mutation, c.1091-2A>G, is one of the most common mutations of *CYP4V2* in Asians, particularly in Chinese and Japanese [8]. It is predicted to cause a skip at exon 9, resulting in the loss of the J'and K helices, which is the critical meander region for heme binding [2]. This structural change may result in the loss of enzymatic activity [2]. The single nucleotide duplication mutation, c.1062dupA, was only detected once in an Indian BCD patient with this single mutation in one allele [14]. This study further supported it as a causative mutation by detecting compound mutations. It is predicted to result in a defective amino acid sequence (p.V355Sfs*4), which might affect heme binding and substrate interactions. By sequencing *TIMP3*, no pathogenic variation was observed, indicating that this CNV may be a potential retinal complication of BCD rather than a coincidental finding. The CNV may be attributed to the chronic disruption of Bruch's membrane and RPE by the crystals.

The patient harboring compound heterozygous mutations c.[802-8_810del17insGC];[1091-2A>G], presented with a lamellar hole and macular cysts in his right eye. Interestingly, no macular hole was detected in his sibling who harbored the same mutations. As Ganesh et al[32] suggested previously, the macular cysts found in this study should be termed as non- cystoid macular edema (non-CME) macular cysts. It differs from CME in patients with retinitis pigmentosa reported previously, since SD-OCT disclosed the presence of cystoid lesions in the macula, but there was no dye accumulation on the FFA, suggesting that vascular leakage plays a minor role in this condition [32]. This is a feature in other reported cases with inherited retinal degenerative diseases, such as juvenile X-linked retinoschisis (XLRS) and NR2E3 retinopathy [32]. It was proposed that macular cysts in these retinal dystrophies might be due to tissue loss secondary to disorganization of retinal architecture in the macular region [32].

In the present study, we found multiple microcystic changes in multiple retinal layers in BCD patients through SD-OCT, a finding consistent with a previous study [7]. However, we suppose that the microcystic changes are attributed to tissue loss presumably secondary to disruption of the retinal architecture by the crystals, rather than subtle macular edema suggested by Garcia-Garcia et al. [7]. Compared to a prevalence from 71% to 100% in the previous study, our SD-OCT study demonstrated ORT only in 3 of the 22 eyes (13.6%) [33], [34], [35]. To the best of our knowledge, this is the first study revealing the presence of ORT in Chinese BCD patients, although BCD is more common in Chinese populations and many cases have been reported [8], [13]. It was suggested that the tubular structures might be due to rearrangement of the degenerating photoreceptors, and other adjacent cells, such as retinal pigment epithelium and glial elements, may also be involved in this process [35]. However, the definite mechanism remains unknown and further studies are needed.

The limitations of this study were the lack of a standard electroretinogram (ERG) or multifocal ERG (mfERG) analysis for the patients. It was previously suggested that ERG is a sensitive indicator of disease progression [3]. Nevertheless, normal ERG amplitude and latency have recently been detected in BCD patients with decreasing visual acuity and night blindness [36]. Therefore, ERG in BCD might not be closely related with disease progression.

In conclusion, we reported the clinical and genetic features in a large number of Chinese patients with BCD, and identified a novel *CYP4V2* mutation c.219T>A (p. F73L) and four known Mutations, c.283G>A (p.G95R), c.802-8_810del17insGC (splicing site), c.1062dupA (p.V355Sfs*4), and c.1091-2A>G (splicing site). Furthermore, our data support the mutation c.219T>A to be another recurrent mutation in Chinese patients with BCD.

## Supporting Information

Table S1 **Summary of** ***CYP4V2*** **mutations**
(DOCX)
