# Supplementary material for: Molecular Analysis and Phenotypic Study in 14 Chinese Families with Bietti Crystalline Dystrophy
Source: PLoS One. 2014 Apr 16;9(4):e94960. doi: 10.1371/journal.pone.0094960 (PMC3989252; doi:10.1371/journal.pone.0094960)
Supplement: Table S1 — Summary of CYP4V2 mutations (DOCX) [file pone.0094960.s001.docx]

| Table S1 Summary of *CYP4V2* mutations | | | | |
| --- | --- | --- | --- | --- |
| No. | Exon | Nucleotide Change | Predicted Protein Change | References |
| 1 | 1 | c.77G >A | p.G26D | [[1](#_ENREF_1)] |
| 2 | 1 | c.130T>A | p.W44R | [[2](#_ENREF_2)] |
| 3 | 1 | c.181G>A | p.G61S | [[2](#_ENREF_2)] |
| 4 | 1 | c.197T>G | p.M66R | [[1](#_ENREF_1)] |
| 5 | IVS1 | c.214+1G>A | Exon1del | [[3](#_ENREF_3)] |
| 6 | IVS1 | c.214+25delT | Not avaliable | [[2](#_ENREF_2)] |
| 7 | IVS1 | c.215-2A>G | Exon2del | [[3](#_ENREF_3)] |
| 8 | IVS1 | c.215-1G>A | Exon2del | [[3](#_ENREF_3)] |
| 9 | 2 | c.219T>A | p.F73L | This study |
| 10 | 2 | c.237G>T | p.E79D | [[2](#_ENREF_2)] |
| 11 | 2 | c.253C>T | p.R85C | [[4](#_ENREF_4)] |
| 12 | 2 | c.277T>C | p.W93R | [[5](#_ENREF_5)] |
| 13 | 2 | c.283G>A | p.G95R | [[4](#_ENREF_4)]; This study |
| 14 | 2 | c.327G>A | Not avaliable | [[2](#_ENREF_2)] |
| 15 | IVS2 | c.327+1G>A | p.E72Gfs*5 | [[2](#_ENREF_2)] |
| 16 | IVS2 | c.327+11G > C | Not avaliable | [[1](#_ENREF_1)] |
| 17 | 3 | c.332T>C | p.I111T | [[2](#_ENREF_2)] |
| 18 | 3 | c.335T>G | p.L112* | [[4](#_ENREF_4)] |
| 19 | 3 | c.367A>G | p.M123V | [[2](#_ENREF_2)] |
| 20 | 3 | c.400G>T | p.G134* | [[2](#_ENREF_2)] |
| 21 | 4 | c.518T>G | p.L173W | [[6](#_ENREF_6)] |
| 22 | 5 | c.637_641delAGTAA | p.S213* | [[7](#_ENREF_7)] |
| 23 | 5 | c.655T>C | p.Y219H | [[8](#_ENREF_8)] |
| 24 | 6 | c.677T>A | p.M226K | [[1](#_ENREF_1)] |
| 25 | 6 | c.694C>T | p.R232* | [[5](#_ENREF_5)] |
| 26 | 6 | c.724delG | p.D242Ifs*35 | [[5](#_ENREF_5)] |
| 27 | 6 | c.732G>A | p.W244* | [[8](#_ENREF_8)] |
| 28 | 6 | c.761A>G | p.H254R | [[3](#_ENREF_3)] |
| 29 | 6 | c.772C>T | p.L258F | [[9](#_ENREF_9)] |
| 30 | 6 | c.775A>C | p.K259Q | [[10](#_ENREF_10)] |
| 31 | 7 | c.802-8_806del13 | Exon7del | [[2](#_ENREF_2)] |
| 32 | 7 | c.802-8_810del17insGC | Exon7del | [[11](#_ENREF_11)]; This study |
| 33 | 7 | c.958C>T | p.R320* | [[3](#_ENREF_3)] |
| 34 | 7 | c.971A>T | p.D324V | [[8](#_ENREF_8)] |
| 35 | 7 | c.974C>T | p.T325I | [[12](#_ENREF_12)] |
| 36 | IVS7 | c.985+3A>G | Not avaliable | [[1](#_ENREF_1)] |
| 37 | 8 | c.992A>C | p.H331P | [[2](#_ENREF_2)] |
| 38 | 8 | c.998C >A | p.T333K | [[1](#_ENREF_1)] |
| 39 | 8 | c.1020G>A | p.W340* | [[11](#_ENREF_11)] |
| 40 | 8 | c.1021T>C | p.S341P | [[2](#_ENREF_2)] |
| 41 | 8 | c.1062dupA | p.V355Sfs*4 | [[13](#_ENREF_13)]; This study |
| 42 | IVS8 | c.1091-2A>G | Exon9del | [[2](#_ENREF_2)]; This study |
| 43 | 9 | c.1157A>C | p.K386T | [[14](#_ENREF_14)] |
| 44 | 9 | c.1168C>T | p.R390C | [[15](#_ENREF_15)] |
| 45 | 9 | c.1169G>A | p.R390H | [[3](#_ENREF_3)] |
| 46 | 9 | c.1187C>T | p.P396L | [[8](#_ENREF_8)] |
| 47 | 9 | c.1198C>T | p.R400C | [[8](#_ENREF_8)] |
| 48 | 9 | c.1199G>A | p.R400H | [[4](#_ENREF_4)] |
| 49 | 10 | c.1226-6_1235del16 | Exon10del | [[3](#_ENREF_3)] |
| 50 | 10 | c.1328G>A | p.R443Q | [[5](#_ENREF_5)] |
| 51 | 10 | c.1348C>T | p.Q450* | [[6](#_ENREF_6)] |
| 52 | 10 | c.1372G>A | p.V458M | [[16](#_ENREF_16)] |
| 53 | 10 | c.1393A>G | p.R465G | [[5](#_ENREF_5)] |
| 54 | 10 | c.1396 A >G | p.N466D | [[17](#_ENREF_17)] |
| 55 | 10 | c.1399T>C | p.C467R | [[18](#_ENREF_18)] |
| 56 | 10 | c.1445C>T | p.S482* | [[14](#_ENREF_14)] |
| 57 | 11 | c.1523G>A | p.R508H | [[2](#_ENREF_2)] |
| 58 | 11 | c.1526C>T | p.P509L | [[19](#_ENREF_19)] |

Numbering is relative to the coding DNA sequence of *CYP4V2* (NM_207352.3), where nucleotide +1 is the A of the ATG translation initiation codon. Mutation is named according to the HGVS Mutation Nomenclature Recommendations (http://www.hgvs.org/mutnomen/recs.html/provided in the public domain by the Human Genome Variation Society).

1. Halford S, Liew G, Mackay DS, Sergouniotis PI, Holt R, et al. (2014) Detailed Phenotypic and Genotypic Characterization of Bietti Crystalline Dystrophy. Ophthalmology.

2. Li A, Jiao X, Munier FL, Schorderet DF, Yao W, et al. (2004) Bietti crystalline corneoretinal dystrophy is caused by mutations in the novel gene CYP4V2. Am J Hum Genet 74: 817-826.

3. Xiao X, Mai G, Li S, Guo X, Zhang Q (2011) Identification of CYP4V2 mutation in 21 families and overview of mutation spectrum in Bietti crystalline corneoretinal dystrophy. Biochem Biophys Res Commun 409: 181-186.

4. Shan M, Dong B, Zhao X, Wang J, Li G, et al. (2005) Novel mutations in the CYP4V2 gene associated with Bietti crystalline corneoretinal dystrophy. Mol Vis 11: 738-743.

5. Rossi S, Testa F, Li A, Yaylacioglu F, Gesualdo C, et al. (2013) Clinical and genetic features in Italian Bietti crystalline dystrophy patients. Br J Ophthalmol 97: 174-179.

6. Lin J, Nishiguchi KM, Nakamura M, Dryja TP, Berson EL, et al. (2005) Recessive mutations in the CYP4V2 gene in East Asian and Middle Eastern patients with Bietti crystalline corneoretinal dystrophy. J Med Genet 42: e38.

7. Manzouri B, Sergouniotis PI, Robson AG, Webster AR, Moore A (2012) Bietti crystalline retinopathy: report of retinal crystal deposition in male adolescent siblings. ARCH OPHTHALMOL 130: 1470-1473.

8. Lai TY, Ng TK, Tam PO, Yam GH, Ngai JW, et al. (2007) Genotype phenotype analysis of Bietti's crystalline dystrophy in patients with CYP4V2 mutations. Invest Ophthalmol Vis Sci 48: 5212-5220.

9. Parravano M, Sciamanna M, Giorno P, Boninfante A, Varano M (2012) Bietti crystalline dystrophy: a morpho-functional evaluation. Doc Ophthalmol 124: 73-77.

10. Bezemer ID, Bare LA, Doggen CJ, Arellano AR, Tong C, et al. (2008) Gene variants associated with deep vein thrombosis. JAMA 299: 1306-1314.

11. Wada Y, Itabashi T, Sato H, Kawamura M, Tada A, et al. (2005) Screening for mutations in CYP4V2 gene in Japanese patients with Bietti's crystalline corneoretinal dystrophy. Am J Ophthalmol 139: 894-899.

12. Zenteno JC, Ayala-Ramirez R, Graue-Wiechers F (2008) Novel CYP4V2 gene mutation in a Mexican patient with Bietti's crystalline corneoretinal dystrophy. Curr Eye Res 33: 313-318.

13. Mamatha G, Umashankar V, Kasinathan N, Krishnan T, Sathyabaarathi R, et al. (2011) Molecular screening of the CYP4V2 gene in Bietti crystalline dystrophy that is associated with choroidal neovascularization. Mol Vis 17: 1970-1977.

14. Lee KY, Koh AH, Aung T, Yong VH, Yeung K, et al. (2005) Characterization of Bietti crystalline dystrophy patients with CYP4V2 mutations. Invest Ophthalmol Vis Sci 46: 3812-3816.

15. Yokoi Y, Sato K, Aoyagi H, Takahashi Y, Yamagami M, et al. (2011) A Novel Compound Heterozygous Mutation in the CYP4V2 Gene in a Japanese Patient with Bietti's Crystalline Corneoretinal Dystrophy. Case Rep Ophthalmol 2: 296-301.

16. Haddad NM, Waked N, Bejjani R, Khoueir Z, Chouery E, et al. (2012) Clinical and molecular findings in three Lebanese families with Bietti crystalline dystrophy: report on a novel mutation. Mol Vis 18: 1182-1188.

17. Fu Q, Wang F, Wang H, Xu F, Zaneveld JE, et al. (2013) Next-generation sequencing-based molecular diagnosis of a Chinese patient cohort with autosomal recessive retinitis pigmentosa. Invest Ophthalmol Vis Sci 54: 4158-4166.

18. Song Y, Mo G, Yin G (2013) A novel mutation in the CYP4V2 gene in a Chinese patient with Bietti's crystalline dystrophy. Int Ophthalmol 33: 269-276.

19. Jin ZB, Ito S, Saito Y, Inoue Y, Yanagi Y, et al. (2006) Clinical and molecular findings in three Japanese patients with crystalline retinopathy. Jpn J Ophthalmol 50: 426-431.
